# Sexual network distribution of HIV self-testing kits: Findings from the process evaluation of an intervention for men who have sex with men in China

**DOI:** 10.1371/journal.pone.0232094

**Published:** 2020-04-22

**Authors:** Wenjing Xiao, Li Yan, Liping Chen, Gengfeng Fu, Haitao Yang, Cui Yang, Hongjing Yan, Chongyi Wei

**Affiliations:** 1 Nanjing Medical University, Nanjing, Jiangsu, China; 2 Xuzhou Central Hospital, Xuzhou, Jiangsu, China; 3 Jiangsu Provincial Center for Disease Control and Prevention, Nanjing, Jiangsu, China; 4 Jiangsu Institute of Parasitic Diseases, Wuxi, Jiangsu, China; 5 Department of Health, Behavior and Society, Johns Hopkins Bloomberg School of Public Health, Baltimore, Maryland, United States of America; 6 Department of Health Behavior, Society, and Policy, Rutgers School of Public Health, New Jersey, United States of America; The Chinese University of Hong Kong, HONG KONG

## Abstract

**Background:**

The World Health Organization has recommended HIV self-testing (HIVST) as an alternative testing strategy given the limitations of facility-based testing. While the benefits of HIV self-testing have been demonstrated at the individual level among men who have sex with men (MSM), limited data exist on if this testing approach can be effectively diffused through individuals’ social or sexual networks. The objectives of this study were to examine patterns and correlates of HIVST distribution within Chinese MSM’s sexual networks.

**Methods:**

Data used for this analysis was a part of the process evaluation of an HIVST intervention trial among MSM in Nanjing, China. Between May and October 2017, we enrolled 400 men into the trial. Participants assigned to the intervention group (N = 200) were given three HIVST kits at baseline and could request more during the follow-up periods. We incorporated measures for process evaluation in the self-administered online follow-up surveys. This analysis reported findings from the three-month follow-up survey in the intervention group. Frequencies and percentages were used to describe characteristics of participants who distributed kits to their sexual partners as well as patterns of distribution. Multivariable logistic regression was conducted to identify independent correlates of participants who distributed the kits.

**Results:**

Of the 177 participants retained (88.5%) at the three-month follow-up, 72 (40.7%) distributed one or more kits to either primary or casual partners. About half of distributors (51.4%) gave one HIVST kit to their sexual partners while 15.3% distributed 3 or more. Over half gave these kits (58.3%) to primary sexual partners while 27.8% reported giving the kits to both primary and casual partners. About half (54.2%) of distributors used the kits together with their partners. Compared to participants who had an HIV test in the past six months, those who tested over six months ago or never tested had significantly lower odds of distributing the kits (AOR = 0.484, 95% CI: 0.250–0.983, p = 0.032). Compared to those who had not used the kits themselves, participants who did had significantly higher odds of distributing the kits (AOR = 3.345, 95% CI: 1.488–7.517, p = 0.003). Participants who reported higher HIV testing efficacy had 2.051 fold greater odds (95% CI: 1.062–3.961, p = 0.033) of distributing the kits compared to those who had lower efficacy.

**Conclusion:**

Our study demonstrated that a sexual network-based approach to distributing HIVST among Chinese MSM is feasible and can be a promising strategy to improve the effectiveness of HIVST programs including its reach to untested men. Such approach should be complimented by intervention components that enhance HIV testing efficacy and improve experiences of HIVST.

## Introduction

According to the Joint United Nations Programme on HIV /AIDS (UNAIDS), by the end of 2017, there were a total of 36.9 million people living with HIV, of which 1.8 million were newly infected [1]. Around the globe, men who have sex with men (MSM) have been disproportionately affected by the pandemic [2, 3]. A meta-analysis reported that MSM were 19.3 times more likely to be infected with HIV than the general population [4].

In China, MSM account for an estimated 28% of new HIV infections annually and is the only key population with increasing new diagnosis despite strengthened efforts on HIV prevention and control in the past decade, including vast expansion of voluntary HIV counseling and testing sites nationwide [5, 6]. Although HIV testing uptake has increased among MSM during the initial periods of expansion, it appears to have plateaued since then and remains suboptimal (e.g., about 50% reported testing in the past year) [7–9]. A recent study found that only 28% of Chinese MSM were willing to get tested for HIV at a local Center for Diseases Control and Prevention (CDC) [10] where free HIV counseling and testing services are offered. By the end of 2015, only 68% of people living with HIV in China were aware of their status [11], significantly below the first target of UNAIDS’ 90-90-90 goal to end the HIV pandemic [12]. Therefore, continuing to improve HIV testing uptake among MSM and other key populations remains a priority.

Recognizing the limitations of facility-based testing (e.g., concerns of privacy, disclosure of stigmatized behaviors), the World Health Organization has officially recommended HIV self-testing (HIVST) as an alternative testing strategy [13]. The recommendation was based on a growing robust evidence base that HIVST is highly acceptable to key populations including MSM, and can significantly increase HIV testing uptake [3, 14–18]. For example, a study of almost 6,000 Chinese MSM reported that almost 40% had ever used HIVST and 92% expressed willingness to use it in the future [18].

While the benefits of HIVST have been demonstrated at the individual level, limited data exist on if this testing approach can be effectively diffused through individuals’ social or sexual networks, which could further enhance its utility and reach. This promise is based on a body of literature showing that network-based HIV testing strategies were effective in reaching MSM in the US, Africa and China and demonstrated evidence for increasing testing among non- and infrequent testers [19]. The primary advantages of network-based strategies include: 1) network members have access to broader networks of MSM who may not access prevention programs or services due to concerns of confidentiality or negative experiences with providers; 2) network members can also access their networks outside clinic hours and in venues often not accessible to traditional outreach workers, overcoming some logistical barriers; and 3) being reached and encouraged to test by peers in their own network whom they trust may alleviate the negative impact of HIV- and gay-related stigma on testing behaviors. In a feasibility study conducted among African American and Latinx MSM in California, it was reported that compared to a local county testing program, the peer-based strategy to distribute HIVST kits through MSM’s social and sexual networks was significantly more likely to reach those who have never tested for HIV and those who were previously undiagnosed [20]. The objectives of this study were to examine patterns and correlates of HIVST distribution within Chinese MSM’s sexual networks.

## Methods

### Study design and recruitment

Data used for this analysis was a part of the process evaluation of an HIVST intervention trial among MSM in Nanjing, China (ClinicalTrials.gov Identifier: NCT02999243). Details of the study design and recruitment have been published elsewhere [21]. Briefly, between May and October 2017, we enrolled 400 men into the trial, who were randomly assigned one-to-one to either the intervention or the control arm. The criteria of eligibility were: 1) being biologically male; 2) being 18 years old or older; 3) being currently residing in Nanjing and planned to stay during the study period; 4) have had oral or anal sex with men in the past year; 5) be confirmed HIV negative by HIV rapid testing. Furthermore, we asked eligible and interested participants to provide their preferred contact information (e.g., WeChat) during the informed consent process for the purpose of follow-up data collection.

We adopted various sampling methods to recruit participants, including online promotion, placement of recruitment advertisements at MSM venues, and referrals from community-based organizations and participants. Interested men could either call the study phone-line or come directly to the study site for an initial eligibility screening. Eligible and consented participants received an HIV rapid test from a trained HIV testing and counseling professional and completed a self-administered electronic questionnaire on a mobile device at baseline. Prior to receiving the test and completing the survey, the study coordinator explained the study to participants in detail, went through the consent process verbally and answered any questions that participants had. Written informed consent was obtained from all participants. Participants assigned to the intervention group were given three HIVST kits (NewScen Coast Bio-Pharmaceutical Co., Ltd., Tianjin, China) to take home with while those in the control group were provided with HIV testing referral information. We did not instruct participants in the intervention group to limit the use of these kits to themselves only. Neither did we actively encourage or instruct participants to distribute kits to their partners since this was not a component of the original intervention design. However, each participant did receive an instructional video for his own use or to share with others. In addition to the three kits provided at baseline, participants in the intervention group could request more during the follow-up periods by contacting our research staff. This was a nine-month intervention trial with a follow-up at every three months. We incorporated measures for process evaluation at the three- and six-months follow-up surveys. Participants completed these surveys online using their own devices. This analysis reported findings from the three-month follow-up survey.

Participants received a 50RMB (1 USD ≈ 6 RMB) pre-paid cellphone card for completing each of the baseline and follow-up surveys. The study was approved by respective Institutional Review Boards at Rutgers University and Jiangsu Provincial Center for Disease Control and Prevention.

### Measures

In the baseline questionnaire, we asked participants of their age, marital status, education, employment status, and sexual orientation. In terms of HIV-related behaviors, we asked participants to report on the recency of their last HIV test, the number of male anal sexual partners in the past six months, and whether or not condoms were used consistently with these male sexual partners when engaging in insertive and/or receptive anal sex. In addition to behaviors, we also included two psychosocial measures relevant to HIV testing: 1) HIV testing efficacy [22] and 2) anticipated HIV stigma [23], both of which have been validated in survey reaserch of Chinese MSM [24]. HIV testing efficacy was measured using a six-item Likert-type scale (e.g., “You can easily discuss HIV testing with potential male sex partners,” “You are still willing to do HIV testing even if you are afraid to know the results.”) (Cronbach’s a = 0.759 in our study participants). Anticipated HIV stigma was measured using a seven-item Likert-type scale (e.g., “I would feel I were not as good a person as others if I got HIV,” “If I got infected men would not want to have sex with me.”) (Cronbach’s a = 0.828 in our study participants). In the analysis, we used the median score as the cut-off for each scale where above the median was considered having higher efficacy and stronger stigma while below the median was considering having lower efficacy and less stigma.

The following process evaluation measures were included in the three-month follow-up questionnaire: If participants have used the HIVST kits themselves; if participants have given any kits to primary or casual male partners; if so, the number of kits given to these partners; whether or not the partners have actually conducted the tests using the kits; if the participants were present when their partners conducted the tests; if the participants showed the instructional video to their partners before using the kits; if the partners were able to use the kits properly; and finally, if the participants have conducted self-testing together with any of these partners (i.e., both used the kits at the same time in the same setting) in the past three months.

### Statistical analysis

Frequencies and percentages were used to describe characteristics of participants who distributed kits to their sexual partners as well as patterns of distribution. Chi-square tests were conducted to compare sociodemographic and behavioral characteristics between participants who distributed kits (distributors) versus those who did not (non-distributors). Variables with significant differences (p ≤ 0.05) in the bivariate analysis were then included in a final multivariable logistic regression model while controlling for age, educational and sexual orientation. All analyses were performed in SPSS version 20.0.

## Results

Of the 200 participants enrolled at baseline and assigned to the intervention arm, 177 (88.5%) were retained at the three-month follow-up. Of these, 124 (70.1%) used the kits themselves and 72 (40.7%) distributed one or more kits to either primary or casual partners in the past three months.

Table 1 presents the socio-demographic characteristics of distributors and patterns of distribution. Most distributors were younger than 35 years old (88.7%) and single (86.1%). A majority had an educational level of college or higher (68.1%), were employed full-time (68.1%), and self-identified as gay (66.7%). About half of distributors (51.4%) gave one HIVST kit to their sexual partners while15.3% distributed 3 or more. Over half gave these kits (58.3%) to primary sexual partners while 27.8% reported giving the kits to both primary and casual partners. Almost all (91.7%) distributors reported that their sexual partners had actually used the kits. While over a half (54.2%) of distributors did not show the instructional video to their partners, 79.2% reported being present while their partners conducted the tests and 19.4% said their partners could not conduct the tests properly. Finally, 54.2% of distributors used the kits together with their sexual partners.

**Table 1:** Socio-demographic characteristics of distributors, and patterns and processes of HIV self-testing (HIVST) distribution among men who have sex with men in Nanjing, China, 2017 (N = 72).

Variables	N (%)
Age	
18–24	30(41.7)
25–34	34(47.2)
> = 35	8(11.1)
Marital status	
Single	62(86.1)
Married/Divorced/Widowed	10(13.9)
Education	
High school or less	8(11.1)
Technical or some college	15(20.8)
College or higher	49(68.1)
Employment	
Full-time	49(68.1)
Student/Part-time/Other	23(31.9)
Sexual orientation	
Gay	48(66.7)
Heterosexual/Not sure	24(33.3)
HIVST kits distributed to	
Primary partner	42(58.3)
Casual partner	10(13.9)
Both	20(27.8)
Number of HIVST kits given to primary or casual partners	
1	37(51.4)
2	24(33.3)
> = 3	11(15.3)
Partners actually used the distributed HIVST kits	
Yes	66(91.7)
No	6(8.3)
Assisted partners to conduct HIVST	
Yes	57(79.2)
No	15(20.8)
Showed partners HIVST instructional video	
Yes	33(45.9)
No	39(54.2)
Any operational errors observed by participants	
Yes	14(19.4)
No/unknown	58(80.6)
Participants and sexual partners conducted HIVST together	
Yes	39(54.2)
No	33(45.8)

Table 2 presents comparisons between distributors and non-distributors at the bivariate level. Compared to non-distributors, distributors were significantly more likely to have had their last HIV test in the past six months (58.3% vs. 36.2%, χ2 = 8.455, p = 0.004), to have used the kits themselves (86.1% vs. 59.0%, χ2 = 14.913, p < 0.001), and to have a higher HIV testing efficacy (56.9% vs. 39.0%, χ2 = 5.502, p = 0.019). There were no significant differences between the distributors and non-distributors in terms of socio-demographics and sexual risk behaviors.

**Table 2:** Bivariate correlates of distributing HIV self-testing (HIVST) kits to sexual partners among men who have sex with men in Nanjing, China 2017 (N = 177).

	Distributors (N = 72)	Non-distributors (N = 105)	χ2	p
Age			2.716	0.257
18–24	30(41.7)	33(31.4)		
25–34	34(47.2)	53(50.5)		
> = 35	8(11.1)	19(18.1)		
Marital status			1.260	0.262
Single	62(86.1)	96(91.4)		
Married/Divorced/Widowed	10(13.9)	9(8.6)		
Education			1.174	0.556
High school or less	8(11.1)	10(9.5)		
Technical or some college	15(20.8)	16(15.2)		
College or higher	49(68.1)	79(75.2)		
Employment			0.818	0.366
Full-time	49(68.1)	78(74.3)		
Student/Part-time/Other	23(31.9)	27(25.7)		
Sexual orientation			1.209	0.272
Gay	48(66.7)	78(74.3)		
Bisexual/Heterosexual/Not sure	24(33.3)	27(25.7)		
Last HIV testing			8.455	0.004
Within 6 months	42(58.3)	38(36.2)		
Over 6 months ago or never tested	30(41.7)	67(63.8)		
Used HIVST kits yourself			14.913	0.000
No	10(13.9)	43(41.0)		
Yes	62(86.1)	62(59.0)		
Number of male anal sex partners			0.228	0.633
≤ 1	30(41.7)	40(38.1)		
≥ 2	42(58.3)	65(61.9)		
Any condomless insertive anal sex			0.622	0.430
No	57(79.2)	88(83.8)		
Yes	15(20.8)	17(16.2)		
Any condomless receptive anal sex			0.001	0.971
No	54(75.0)	79(75.2)		
Yes	18(25.0)	26(24.8)		
HIV testing efficacy			5.502	0.019
Low	31(43.1)	64(61.0)		
High	41(56.9)	41(39.0)		
Anticipated HIV stigma			0.000	0.983
Low	44(61.1)	64(61.0)		
High	28(38.9)	41(39.0)		

Table 3 presents multivariable correlates of being a distributor. After controlling for age, education and sexual orientation, compared to participants who had an HIV test in the past six months, those who tested over six months ago or never tested had significantly lower odds of distributing HIVST kits to sexual partners (AOR = 0.484, 95% CI: 0.250–0.983, p = 0.032). In addition, compared to those who had not used the kits themselves, participants who had used the kits had significantly higher odds of distributing the kits to sexual partners (AOR = 3.345, 95% CI: 1.488–7.517, p = 0.003). Finally, participants who reported higher HIV testing efficacy had 2.051 fold greater odds (95% CI: 1.062–3.961, p = 0.033) of distributing the kits to their partners compared to those who had lower efficacy.

**Table 3:** Multivariable correlates of distributing HIV self-testing (HIVST) kits to sexual partners among men who have sex with men in Nanjing, China 2017 (N = 177).

Variables	AOR (95% CI)	*p*
Age		0.225
18–24	1	
25–34	0.645(0.311,1.340)	
> = 35	0.413(0.141,1.212)	
Education		0.923
High school or less	1	
Technical or some college	1.168(0.326,4.183)	
College or higher	0.978(0.320,2.986)	
Sexual orientation		0.463
Gay	1	
Bisexual/Heterosexual/Not sure	1.313(0.635,2.714)	
Last HIV testing		
Within 6 months	1	0.032
Over 6 months ago or never tested	0.484(0.250,0.938)	
Used HIVST kits yourself		
No	1	0.003
Yes	3.345(1.488,7.517)	
HIV testing efficacy		
Low	1	0.033
High	2.051(1.062,3.961)	

## Discussion

In this study, we examine patterns and correlates of HIVST distribution within Chinese MSM’s sexual networks. We found that a substantial number of MSM participants distributed HIVST kits to their male sexual partners, and among these about a half reported using the kits together with their partners. As reported in many studies that HIVST was highly acceptable, in particular in contexts where stigma was prevalent [14, 25, 26], our findings also suggest that a sexual network-based strategy of distributing HIVST kits is a feasible and promising approach to reach Chinese MSM who may not seek HIV testing on their own. This is consistent with findings from other studies conducted among MSM in the US and South Africa [16, 20]. In addition, when provided with the right tool, it may encourage couples in primary or casual relationships to seek awareness of their mutual HIV status and thus inform decisions on sexual practices. China’s most recent Five-Year Action Plan for HIV/AIDS Prevention has included HIVST as one of the testing tools to increase access to HIV testing among those who have never tested [27]. This study provides further evidence for health authorities at local, provincial and national levels to implement the strategy.

We also found that participants who had used the HIVST kits themselves had significantly higher odds of distributing the kits to sex partners. This suggests that men who had conducted HIVST themselves may be satisfied with this testing tool and were then more likely to share it with their partners. In addition, participants not only distributed the kits to their primary sexual partners, but also to their casual sexual partners. As many casual relationships occur in a fast fashion mode (e.g., hookups on social networking mobile applications), getting tested at a facility before the sexual encounter is an unlikely option. HIVST offers the convenience of testing for men who want to confirm of each other’s HIV status privately and quickly. Not surprising, men who reported higher HIV testing efficacy were more likely to distribute HIVST kits to sexual partners. In order to further improve efficiency of HIVST diffusion through MSM’s sexual networks, intervention components that increase efficacy should be incorporated into network-based HIVST implementation strategies.

In this study, HIVST was conducted using finger prick modality and a majority of participants reported assisting their partners during the HIVST process. While a majority also reported that no errors were observed during this process suggesting that the finger prick modality was relatively easy to perform, nearly one in five participants reported that their partners made some error which is consistent with previous research [28]. This could more likely results in testing false negative, leading some men to engage in high risk behaviors and unknowingly transmit HIV to others [29]. Therefore, developing standardized multi-media HIVST instructions and providing professional counseling in real time through telemedicine approaches could enhance HIVST experiences and facilitate its expansion.

Several limitations of this study should be noted. First, our findings may not be generalizable to MSM in other areas of China in particular non-urban settings. However, this was among the first few studies to provide insights into network-based approaches to HIVST distribution among Chinese MSM. Second, as a pilot intervention, our sample size was relatively small, possibly limiting power to detect some differences (e.g., sexual risk behaviors). However, the foci of the process evaluation was to describing patterns and processes of sexual network distribution of HIVST. Third, there might be social desirability bias. However, the online survey was self-administered and anonymous. Fourth, this analysis only described the process evaluation at the three-month follow-up. During this relatively short period of time, some MSM may not have had sexual partners or not have changed their partners, leading to an underestimate of distribution rates among participants. However, the three-month follow-up period may reduce recall bias and we did find that the distribution rate was substantial. Finally, to reduce burden for participants, we did not include a detailed matrix to measure sexual behaviors, limiting our ability to observe other nuances of distributing patterns.

## Conclusions

HIVST has been proven efficacious to improve HIV testing uptake among key populations including MSM in controlled trials and recommended by WHO as an alternative HIV testing approach. To maximize its effectiveness in real world settings is the next logical step. Our study demonstrated that a sexual network-based approach to distributing HIVST among Chinese MSM is feasible and can be a promising strategy to improve the effectiveness of HIVST programs including its reach to untested men. Such network-based approach should also be complimented by intervention components that enhance HIV testing efficacy and improve experiences of HIVST.

## Supporting information

S1 Data Deidentified survey data. (XLSX)
